# Reduced cutout for reverse oblique intertrochanteric hip fractures treated with trochanteric fixation advanced (TFN-A) nail compared to the short gamma-3 nail

**DOI:** 10.1051/sicotj/2023013

**Published:** 2023-06-05

**Authors:** Etay Elbaz, Samuel Morgan, Shai Factor, Or Shaked, Nadav Graif, Tomer Ben-Tov, Amal Khoury, Yaniv Warschawski

**Affiliations:** 1 Division of Orthopedics, Tel Aviv Sourasky Medical Center, Tel-Aviv, Affiliated to the Sackler Faculty of Medicine Tel Aviv University Tel Aviv 6423906 Israel; 2 Sackler Faculty of Medicine, Tel Aviv University Tel Aviv 6423906 Israel

**Keywords:** Reverse oblique fracture, TFNA nail, Gamma 3 nail

## Abstract

*Background*: Reverse oblique fractures (AO/OTA 31-A3) account for 5–23% of all intertrochanteric fractures and are challenging to manage. The Gamma 3-Proximal Femoral Nail (GPFN) and the Trochanteric Fixation Nail Advanced (TFNA) are two common cephalomedullary systems used to treat this fracture. No study has reported on outcomes with the TFN-A for reverse oblique fractures. This study aimed to compare outcomes and complication rates in patients with reverse oblique fractures, treated with either TFNA or GPFN. *Patients and methods*: A total of 203 patients with reverse oblique fractures (137 in the GPFN group and 66 in the TFNA group), were treated in our institution between June 2010 and May 2019. Data was collected on postoperative radiological variables including screw or blade location, and tip-apex distance (TAD). Data were also collected for non-orthopaedic complication rates and orthopaedic complications. A sub-group analysis was additionally performed for different nail lengths. *Results*: We found no significant difference in the overall rate of complications and revisions between the two groups. Patients treated with the 235 mm TFN-A nail sustained lower rates of cutout, compared to 180 mm GPFN (GPFN: 6% TFN-A: 0%, *p* = 0.043). The frequency of revision surgeries and malunions/non-unions did not differ significantly between the two groups and additionally showed no difference in the subgroup analysis. *Conclusion*: The 235 mm TFN-A was associated with lower rates of cut-out compared to the short GPFN for reverse oblique intertrochanteric fractures. Future well-designed prospective studies are warranted to investigate the role of the TFN-A in improving outcomes for such fractures.

## Introduction

Hip fractures are increasing in incidence and are a significant source of morbidity and mortality for elderly patients [1–3]. Reverse oblique fractures are extracapsular hip fractures, classified as AO/OTA 31-A3, according to the Trauma Association classification system, and account for 5–23% of all intertrochanteric fractures [3, 4]. These fractures are characterized by a line that extends from the proximal-medial to the distal-lateral region, through the intertrochanteric-subtrochanteric region. Such a pattern results in compromise to the lateral trochanteric wall, making these fractures highly unstable and consequently, very challenging to achieve stable fixation [4]. Despite the relatively low incidence of these fractures, the incidence of implant failure is high, compared to AO/OTA 31-A1 and A2 fractures [5, 6].

The treatment of 31-A3 fractures is controversial, however, there is a growing body of evidence supporting the use of cephalomedullary fixation [7–10]. Two of the most common cephalomedullary systems used in orthopaedic departments include the Trochanteric Fixation Nail Advanced (TFNA; DePuy Synthes) and the Gamma 3 Proximal Femoral Nail (GPFN; Stryker) [5, 6]. Compared to the GPFN, the TFNA nail is a newer implant, intended to represent a step up from its contemporary nails. Its titanium alloy material is purported to enhance fatigue strength and its proximal nail geometry is better suited to fit the anatomic bow of the femur [11]. Its novel design may serve beneficial for the fixation of highly unstable 31-A3 fractures. Despite its aforementioned advantages, the introduction of the TFNA implant has provoked scrutiny among members of the orthopaedic community due to a case series that described a potentially novel mode of failure [12]. Moreover, a recent study that compared data from 342 implant failures between the TFN-A and gamma-3 implants, reported a shorter time to failure associated with the TFNA nail [7].

There is currently a paucity of evidence in orthopaedic literature comparing outcomes between nails used to manage reverse oblique hip fractures [9, 13]. Due to the complexity of management and the high risk that these fractures represent, improvements in our understanding of implant performance for these fractures are essential. Studies reporting outcomes of reverse oblique fractures managed with the TFNA nail are warranted in order to determine if the following system has a role to play in the management of these fractures, with unique biomechanical characteristics.

Thus, the purpose of the present study was to compare the clinical outcomes of the TFNA implant with the outcomes of the GPFN for the treatment of reverse oblique fractures. To the best of our knowledge, no study has previously compared the TFNA device to any method of fixation for reverse oblique fractures.

## Materials and methods

After receiving local ethics committee approval, data were retrieved from the medical records of consecutive patients that underwent closed reduction and internal fixation (CRIF) for AO/OTA 31-A3 fractures between June 2010 and May 2019. The implantation of the TFNA nail began in our institution on March 2017. Therefore, patients who received a GPFN nail were operated on from June 2010 to February 2017, and all patients who received a TFNA nail were operated on from March 2017 to May 2019.

Patient demographics were collected from the patient’s electronic medical records and consisted of the following: gender, age, and ASA (American Society of Anesthesiology) score. Excluded from our study were patients with a pathological fracture, patients who underwent revision surgery and patients who did not meet the minimum 1-year follow-up period required.

All patients underwent CRIF within 48 h of presentation to our level 1 trauma centre. Patients were operated on under general or regional anaesthesia and positioned supine on a fracture table. Fracture reduction with the rotational restoration was completed under fluoroscopy. Operational data such as the surgeons’ level of experience, duration of surgery, and decreased haemoglobin levels, together with data on other hospitalisation characteristics and any intraoperative and postoperative complications were retrieved from the medical files.

Postoperative management included early mobilization, full-weight bearing, and preventive prophylactic treatment for thromboembolism, with daily subcutaneous injection of Enoxaparin sodium (brand name Lovenox™) of 40 mg for 30 days.

Patients were routinely examined at our outpatient clinic at 3 weeks, 6 weeks, 3 months, 6 months and one year postoperatively. Radiological evaluation of AP and axial films were performed by a senior surgeon. The Tip to Apex Distance (TAD) was measured as the sum of the distance from the tip of the blade (i.e. blade or screw) to the apex of the femoral head on AP and lateral views. In addition, malunion was defined by more than 10 degrees of varus or valgus compared with the unaffected hip and more than 10 mm of shortening. Non-union was defined as either no callus or with a callus that did not bridge the fracture site at least 15 weeks after the fracture [13, 14].

## Statistical analysis

Statistical analysis was performed with SPSS version 25 (IBM SPSS Statistics, Chicago, IL, USA). Descriptive statistics were applied for patient characteristics. After continuous variables were tested for normality, means and standard deviations (SD) were calculated, and compared by Student’s *t*-test. Frequencies and percentages were calculated for nominal variables and were compared between the groups by the Chi-square test. Differences between study groups were considered statistically significant at *p* < 0.05.

## Results

The final study population consisted of 203 patients, that met the inclusion criteria, with one hundred and thirty-seven patients in the GPFN group and 66 patients in the TFNA group. There was no difference between the groups in terms of age (*p* = 0.942), gender (*p* = 0.541), and Charlson score (*p* = 0.876) (Table 1).

**Table 1:** Patients and surgical characteristics.

	GPFN (*n* = 144)	TFNA (*n* = 66)	*p*-value
Age, (SD)	82.18 (10.3)	82.06 (11.0)	0.942
Gender, *n* (%)			
Female	107 (78.1)	49 (74.2)	0.541
Charlson score	5.20 (2.0)	5.15 (2.2)	0.876
Side, *n* (%)			
Right	69 (50.4)	28 (42.4)	0.289
Time to surgery, days (SD)	1.34 (1.5)	1.19 (1.1)	0.502
Blood loss, g/dL (SD)	2.8 (1.5)	2.7 (1.4)	0.529
Length of surgery, min (SD)	64.6 (29.3)	70.5 (39.3)	0.236
Surgeon, *n* (%)			
Resident	85 (62.0)	36 (54.5)	0.308
AP peg, *n* (%)			
Middle	51 (37.2)	36 (54.5)	
Superior	4 (2.9)	0 (0)	
Anterior	26 (19.0)	3 (4.5)	<0.001
Axial peg, *n* (%)			
Middle	81 (59.1)	31 (47.0)	
Posterior	30 (21.9)	32 (48.5)	
TAD, mm (SD)	22.5 (6.5)	21.8 (7.2)	0.476
Subclassification, *n* (%)			
31-A3.1	48 (35.6)	31 (47.0)	0.001
31-A3.2	21 (15.6)	20 (30.3)	
31-A3.3	66 (48.9)	15 (22.7)	
180 mm	100 (73)	0 (0)	
235 mm	0 (0)	66 (100)	
Long nail (>235 mm)	37 (27)		
Medical complications, *n* (%)	32 (23.4)	14 (21.2)	0.732
Revisions, *n* (%)	14 (10.2)	3 (4.5)	0.172
Follow-up, months (SD)	18.1 (18.9)	12.1(8.6)	<0.001
ASA score, *n* (%)			
1	13 (16.0)	3 (5.3)	0.144
2	28 (34.6)	21 (36.8)	
3	39 (48.1)	30 (52.6)	
4	1 (1.2)	3 (5.3)	
Orthopaedic complications, *n* (%)	18 (13.1)	13 (19.7)	0.224

The GPFN group age range was 28–96 years (mean 82.1 ± 10.3 years), with 78.1% females, while the TFNA group age range was 30–102 (mean 82.06 ± 11 years), with 74.2% being females.

No significant difference was noted in terms of post-operative orthopaedic complications between the GPFN group and the TFNA group, 18 cases versus 13 cases respectively, (*p* = 0.224). The frequency of revision surgeries did not differ significantly between the two groups (14 GPFN vs. 4 TFNA, *p* = 0.273). The mean TAD in the TFNA group was 21.8 mm, while the average TAD in the GPFN group was 22.5 mm (*p* = 476). The main orthopaedic complication in the GPFN group was the cutout of the peg from the femoral head, which occurred in 7 cases (4.89%), compared to 0 cases in the TFNA group.

Further analysis, comparing the different outcomes regarding the length of the nail, showed 6 cutouts in the short GPFN group (6%) compared to no cutouts in the 235 mm length TFNA nail group (*p* = 0.043) (Table 2).

**Table 2:** Surgical outcome comparison between different lengths of nails.

	TFNA 235 mm^a^ (*n* = 66)	Short GPFN^b^ (*n* = 100)	*p*-value
Cutouts, *n* (%)	0 (0)	6 (6.0)	0.043
Malunion & nonunion, *n* (%)	1 (1.5)	2 (2.0)	0.818
Revisions, *n* (%)	3 (4.5)	10 (10.0)	0.2
	TFNA 235 mm (*n* = 66)	Long GPFN^c^ (*n* = 37)	
Cutouts, *n* (%)	0 (0)	1 (2.7)	0.18
Malunion & nonunion, *n* (%)	1 (1.5)	2 (5.4)	0.26
Revisions, *n* (%)	3 (4.5)	4 (10.8)	0.225
	Short GPFN (*n* = 100)	Long GPFN (*n* = 37)	
Cutouts, *n* (%)	6 (6.0)	1 (2.7)	0.436
Malunion & nonunion, *n* (%)	2 (2.0)	2 (5.4)	0.293
Revisions, *n* (%)	10 (10.0)	4 (10.8)	0.889

a TFNA 235 mm long.

b GPFN 170–220 mm long.

c GPFN longer than 220 mm.

## Discussion

The purpose of the present study was to compare the outcomes for reverse oblique intertrochanteric fractures treated with the GPFN and TFNA implant systems. We found no significant difference in the overall rate of complications and revisions between the two groups. We performed an additional subgroup analysis where we compared the short and long GPFN to the TFNA and notably, found that patients treated with the 235 mm TFN-A nail sustained significantly lower rates of cut-out, compared to the short GPFN group.

Reverse oblique fractures are highly unstable and have a tendency for the femoral shaft to displace medially [13]. The optimal device for such a fracture would resist the medialization of the femoral shaft, while also resisting varus angulation of the proximal fragment that is caused by forces from the hip abductors [14]. While controversy exists surrounding the treatment of this fracture, a large body of evidence supports cephalomedullary fixation. [7–10] Intramedullary fixation has additionally been shown to be a cost-effective option for 31-A3 fractures [15].

The most notable finding in our study was the lower rate of cut-out observed for the TFN-A group, compared to the short GPFN. However, no difference was noted in the cut-out when comparing the long GPFN to the TFN-A. Cut-out which occurs due to the collapse of the neck-shaft angle into varus is more likely to occur with A3 fractures that are difficult to reduce [6]. We believe that nail length is an important factor which may have contributed to the differing results across subgroups. Irgit et al. [16] reported 1 cutout in 148 consecutive AO 31-A3 fractures treated with long cephalomedullary nails. The authors additionally proposed that an ideal TAD < 25 may not be as significant for reverse oblique fractures treated with cephalomedullary nails. Our findings agree with this notion regarding the TAD for reverse oblique fractures, as our TAD showed no difference between the groups with an average of 22.5 mm (6.5) and 21.9 mm (7.2), for the GPFN and the TFNA, respectively, (*p* = 0.476). Overall, the rate of cut-out across all nails in our study was relatively low and was in agreeance with rates reported in the literature [17–21].

Previous studies have recommended the implantation of long nails for the treatment of reverse oblique fractures [22, 23]. Our results corroborate such findings and suggest that for reverse oblique fractures, short nails, which are less stable may be more predisposed to cut-out. Conversely, Okcu et al. [24] found no significant difference in the overall complication rate for the treatment of reverse oblique fractures between long nails (>34 cm) and standard nails (24 cm). A recent biomechanical analysis by Blum et al. [25] additionally suggested no benefit associated with long nails for the treatment of unstable trochanteric fractures. The current evidence on this topic is inconclusive and further detailed investigation is required.

The material of the nails is a potential source that could explain the discrepancy in our results as it pertains to cut-out. The TFNA nail was introduced, as having different properties in material, length, variety, and design. A series of laboratory fatigue tests revealed it to be 24% stronger than its contemporary Gamma-3 nail and 47% stronger than the Intertan nail [11]. Its enhanced fatigue resistance and strength are thought to be derived from its titanium alloy material [6, 16]. Little is known regarding the performance of this nail for reverse oblique hip fractures. Our results add to limited evidence and suggest it has comparable outcomes to its contemporary cephalomedullary nails. Further investigation is warranted in order to determine if this implant reduces cut-outs in these unstable fractures.

We observed relatively low revision rates for both nails in our study. In a previous study of ours, where we compared the expandable and gamma proximal femoral nails, 11/40 GPFNs (27.5%) were revised following the treatment of reverse oblique fractures [8]. We believe this may in part be caused by the small sample size (*n* = 40) used in that study. Bonnaire et al. [9] similarly reported a revision rate of 7.5% in the current study, in unstable trochanteric fractures managed with GPFN. The lower rate of revision in their study could be attributed to the fact that they considered AO/OTA 31-A2 fractures unstable.

In the current study, our blade and screw position was appropriate in the AP and axial views with no significant differences between the two groups. Hundred percent of the TFNA and 97.2% of the GPFN were middle-inferior in the AP view. Ninety-six percent and eighty-two percent were in the middle-posterior position in the axial view for the TFNA and GPFN groups, respectively. Such findings suggest that in both cohorts, the peg position was acceptable. However, from the 6 cases of the cutout that occurred in the short GPFN group, the peg was anterior in the axial view in 67% of them. Such a factor may predispose to cut out and should be avoided when possible.

The current study is not without limitations. First, this is a retrospective design. The two groups were not equal in their sample size, with the GPFN group almost double in size, which had the potential to confound findings. Second, this study was solely focused on reverse oblique intertrochanteric fractures. As these fractures are more challenging to manage compared to A1 and A2 fractures, the complication rate is naturally higher in both groups. Third, surgeon experience is a potential factor that could have biased our results as 62% of the surgeries in the GPFN group were performed by residents compared to 54% in the TFN-A group. We acknowledge that when managing these difficult fractures, resident level (ie R2 vs. R4) and type of surgical assist (i.e. attending versus other trainees) is a notable factors that can impact results. As we did not stratify residents according to their level of training nor identify the level of assistance, this poses an additional limitation. Fourth, the TFNA group had a 235 mm length (95.9%), whereas only 26.6% of the nails in the GPFN group were long (defined as >220 mm in length). Thus, nail length could have confounded findings.

Despite the aforementioned limitations, our findings contribute evidence to the management of unstable reverse oblique fractures. Future well-designed larger prospective studies are warranted to determine the safety and cost-effectiveness of utilizing both implant systems in the management of 31-A3 fractures.

## Conclusion

To the authors knowledge, this study is the first to directly compare outcomes of treating reverse oblique intertrochanteric fractures (AO/OTA 31-A3 classification) with GPFN and TFNA. As we observed comparable complication rates between the GPFN and TFN-A, surgeons may readily consider the 235 mm TFN-A in the management of these unstable fractures. Compared to the short GPFN, the TFN-A was associated with lower cut-out, suggesting this nail may be beneficial in this regard. Nail length should serve as an important consideration when selecting an implant for such fractures. Further detailed investigation with larger sample sizes and prospective nature should be conducted in order to better understand the performances of different implant systems for this unstable fracture with unique biomechanical characteristics.
